# Ready for testing artificial intelligence in radiology clinical practice: We would do well to be in the front line leveraging their strengths but also highlighting today weaknesses

**DOI:** 10.1007/s00330-023-10240-y

**Published:** 2023-09-22

**Authors:** Benjamin Bender

**Affiliations:** Department of Diagnostic and Interventional Neuroradiology, Radiologic Clinics, University Hospital Tübingen, Hoppe-Seyler-Str. 3, 72076 Tübingen, Germany

The workload of radiologists has seen relentless growth in recent years [1]. While the increase of raw image data is not linearly linked to increased reading time, and there is no credible relationship between the speed of diagnostic image interpretation and accuracy [2], many argue that artificial intelligence (AI) could become a helpful tool to aid radiologists [3] or even eliminate the need for radiologists [4].

Deep learning as a specific strategy of machine learning in artificial intelligence has become a valuable tool in scientific research in recent years. It uses artificial neural networks (ANNs) to identify patterns or features in large datasets, based on the principle of how physiological neurons are believed to interact with each other. Consider the retina and its neuronal wiring as an analogy for an ANN. Large amounts of information are used as an input (first layer; in the retina, the rod and cone cells) and the information is condensed over two layers (bipolar and ganglion cells) with decreasing number of cells in each layer. Based on the activation state (on/off) and the type of connection of the cells of the upper layer, the state of the cell in the next layer is changed. In the visual cortex, this information is then dispersed again, to an increasing number of cells in the primary and secondary visual centers. In the end, the human brain creates an interpretation of what it sees, which can be a variety of different types of information, such as a classification (e.g., is it a car or a flower) or a segmentation (e.g., leaves and blossoms of a flower). During the training of an ANN, the computer tries to optimize the interactions of the artificial neurons in each layer (how a certain state will affect the next layer) in order to achieve the set goal.

Since deep learning achieves astonishing results, especially in typical “visual” tasks, it is very well suited to radiology. However, the ANN’s performance strongly depends on how it was trained. This requires a large set of training data tailored to the ANN’s task, which must usually be labeled by hand. For this reason, high-volume radiological examinations are a prime application for AI, thanks to the large amount of high-quality data are available to train and validate algorithms [3].

Bucklak et al [5] used an exceptionally large and heterogenous dataset of over 210,000 non-contract computed tomography scans of the brain in almost 170,000 unique patients to train ANNs to detect 192 findings summarized in 22 parent findings. They made a tremendous effort to manually label all the datasets and to double check the datasets used for testing by a subspecialized neuroradiologist. Even with this large dataset, the output from 48 findings had to be excluded, as the ANN did not reach the performance expected. Three additional findings were excluded from statistical evaluation due to the low number of test cases. These challenges do not limit the relevance of the results. Indeed, they highlight the problems we still face despite the technical possibilities of AI, and the need for and value of large, well-labeled datasets.

The authors performed a large-scale performance test with 32 radiologists. Each radiologist interpreted 2848 test cases (not used in the training of the algorithm) and rated the presence (yes/no) of the 192 findings once without and once with the information from the 144 adequately performing ANN findings.

On its own, the ANN model performed better than the average radiologist performance on the subset of 144 findings included in the model. Moreover, overall performance of the radiologist on correct detection of findings increased when assisted by the model. Using the Matthews correlation coefficient to evaluate performance at a given threshold, which is a more realistic description of deciding whether a finding is present or not, reader performance was statistically significantly improved in 81 findings.

Interestingly, despite superior model performance, a decrease in AUC within the 95% CI was still detected in the assisted evaluation in 17 findings, many of which were not reported by the model. While the decrease was above the defined threshold of clinical significance, one must keep in mind that the use of a support system might decrease the attention to unreported findings in a clinical setting. This is especially relevant as rare but critical diseases like basilar thrombosis, encephalitis, or venous sinus thrombosis were excluded from the AI output due to model performance.

The authors nicely described the possible benefits of AI models in radiological practice, where a support system can increase confidence and guide interpretation of images as it helps to direct focus. This could be especially relevant in off-hours in which radiologic residents provide preliminary interpretations, though clinical relevance has yet to be proved as misinterpretations requiring a change in clinical management are rare [6]. Nevertheless, AI cannot currently replace a radiologist as suggested by Lexa et al [4] or a good radiological education, since rare but potential vital findings are not directly supported, and differential diagnosis also relies on clinical information.

Bucklak et al [5] also point out the possible risks of AI tools like automation bias. These risks can be minimized by educated users, and radiologists would do well to engage with these emerging technologies, evaluate them in clinical practice, leverage their strengths but also highlight their current weaknesses. As the AI tool evaluated here is certified in many regions (FDA, MDR, Australia, New Zealand, UK, Singapore), it can be readily integrated into clinical practice. An obstacle could be that in many countries there are no reimbursement possibilities for the application, which means additional costs.

In summary, the work of Bucklak et al [5] demonstrates the possibility that AI tools can improve the quality of radiological reports. At a time of ever-increasing workload, it can be understood as a call to accelerate the introduction of AI tools in the radiological routine and to guide their use scientifically, to validate the benefit not only retrospectively as shown here, but also prospectively and with more clinically meaningful endpoints [7] such as time till treatment onset or clinical scores, like 90-day mRS in stroke.

## Declarations

### Guarantor

The scientific guarantor of this publication is Benjamin Bender.

### Statistics and biometry

No complex statistical methods were necessary for this paper.

### Methodology

•commentary
